# Human cerebral organoids as a therapeutic drug screening model for Creutzfeldt–Jakob disease

**DOI:** 10.1038/s41598-021-84689-6

**Published:** 2021-03-09

**Authors:** Bradley R. Groveman, Natalia C. Ferreira, Simote T. Foliaki, Ryan O. Walters, Clayton W. Winkler, Brent Race, Andrew G. Hughson, Gianluigi Zanusso, Cathryn L. Haigh

**Affiliations:** 1 Laboratory of Persistent Viral Diseases, Division of Intramural Research, Rocky Mountain Laboratories, National Institute of Allergy and Infectious Diseases, National Institutes of Health, Hamilton, MT 59840 USA; 2 Department of Neurosciences, Biomedicine and Movement Sciences, University of Verona, 37134 Verona, Italy

**Keywords:** Biochemistry, Drug discovery, Molecular biology, Neuroscience, Biomarkers, Diseases, Medical research, Molecular medicine, Pathogenesis

## Abstract

Creutzfeldt–Jakob Disease (CJD) is a fatal, currently incurable, neurodegenerative disease. The search for candidate treatments would be greatly facilitated by the availability of human cell-based models of prion disease. Recently, an induced pluripotent stem cell derived human cerebral organoid model was shown to take up and propagate human CJD prions. This model offers new opportunities to screen drug candidates for the treatment of human prion diseases in an entirely human genetic background. Here we provide the first evidence that human cerebral organoids can be a viable model for CJD drug screening by using an established anti-prion compound, pentosan polysulfate (PPS). PPS delayed prion propagation in a prophylactic-like treatment paradigm and also alleviated propagation when applied following establishment of infection in a therapeutic-like treatment paradigm. This study demonstrates the utility of cerebral organoids as the first human 3D cell culture system for screening therapeutic drug candidates for human prion diseases.

## Introduction

Prion diseases are fatal neurodegenerative diseases which arise after the conformational conversion of native prion protein (PrP^C^) to the misfolded and pathogenic form, termed PrP^Sc^^1–4^. The existence of different PrP^Sc^ conformations gives rise to prion strains. In sporadic CJD, prion strains or molecular subtypes differ from each other biochemically and this can be distinguished using PrP electrophoretic mobility, proteolytic resistance and glycosylation pattern (un-, mono-, or di-glycosylated^5^). A polymorphism at amino acid residue 129, which may be a methionine or valine^6^, also influences subtype incubation period, clinical signs, and neuropathology^7^. The prion strain phenomenon is extremely important when developing prion therapeutics, as a given drug can show effectiveness for a certain strain and be ineffective against others^8^. Etiologically, the PrP misfolding process can occur (i) spontaneously (sporadic form), (ii) be triggered by unstable PrP, which is generated by inherited genetic mutations in the prion protein gene or (iii) acquired through exposure to PrP^Sc^^9–12^. Regardless of its origin, once present, PrP^Sc^ is able to induce the conversion of PrP^C^ monomers into the misfolded form in a self-propagating manner generating insoluble aggregates^13,14^. The accumulation of these aggregates is followed by neuronal death, astrocytosis and brain damage, leading to dementia or movement disorders. Despite its long incubation period, which can vary from years to decades, there is no therapeutic intervention approved or available that is able to stop or slow prion disease progression, and the patient usually dies within a year of symptom onset^15^. This has instigated a search for therapeutics to treat prion diseases lasting three decades with no successful treatment available to date^15^.

Different models have been used over the years in attempts to identify anti-prion drugs^16,17^ including animal bioassays^18^, immortalized cell lines^19–24^, and cell-free conversion assays^14,25^. However, no reproducible model for studying human prion infection had been reported until recently when human induced pluripotent stem cell (iPSC) derived astrocytes^26^ and human cerebral organoids (COs)^27^ were successfully infected with sCJD. Over the last few years, COs have been widely used to study a variety of brain disorders, such as microcephaly^28,29^, Parkinson’s^30^, and Alzheimer’s diseases^31^. The CO model for sCJD represents the most complete human (cell or tissue) model in the prion field and opens the door to a new platform for drug discovery.

Organoids have previously been shown to accurately predict neurotoxicity of drug treatments in humans^32^, and conversely can be used to predict the safety of a given compound. Furthermore, patient specific organoids have been shown to be good predictors of a patient’s responsiveness to medications. In patients with cystic fibrosis, organoids were successfully used to identify patients that would be responsive to specific drug treatments^33^. This suggests that it is far more likely that a candidate identified using the CO model will be effective in humans than using other models. Prion inhibitors identified through in vitro assays using mice infected with mouse prion strains are usually effective at extending the survival time in these bioassays. However, when using the more relevant animal models expressing human PrP^C^ infected with CJD prions, the same inhibitors often fail to show significant effects^8,34–36^. As previously stated, no anti-prion compounds have ever been effective in humans, however pentosan polysulfate (PPS) is an established anti-prion compound, widely known for its ability to inhibit prion propagation in cell culture^37^. PPS has also been demonstrated to extend survival times in prion-infected mice treated prophylactically^38^ or therapeutically early in disease course^39,40^. The ultimate failure of this molecule in humans occurs due to its lack of blood brain barrier permeability and the requirement of delivery intra-cranially^41–43^. As such it is only suitable for patient use on compassionate grounds at a time of disease where the damage may be irreversible. In this study we used PPS to demonstrate the utility of sCJD infected COs for evaluating therapeutics for prion disease.

## Results

### Prophylactic treatment of COs with PPS

**Figure 1:**
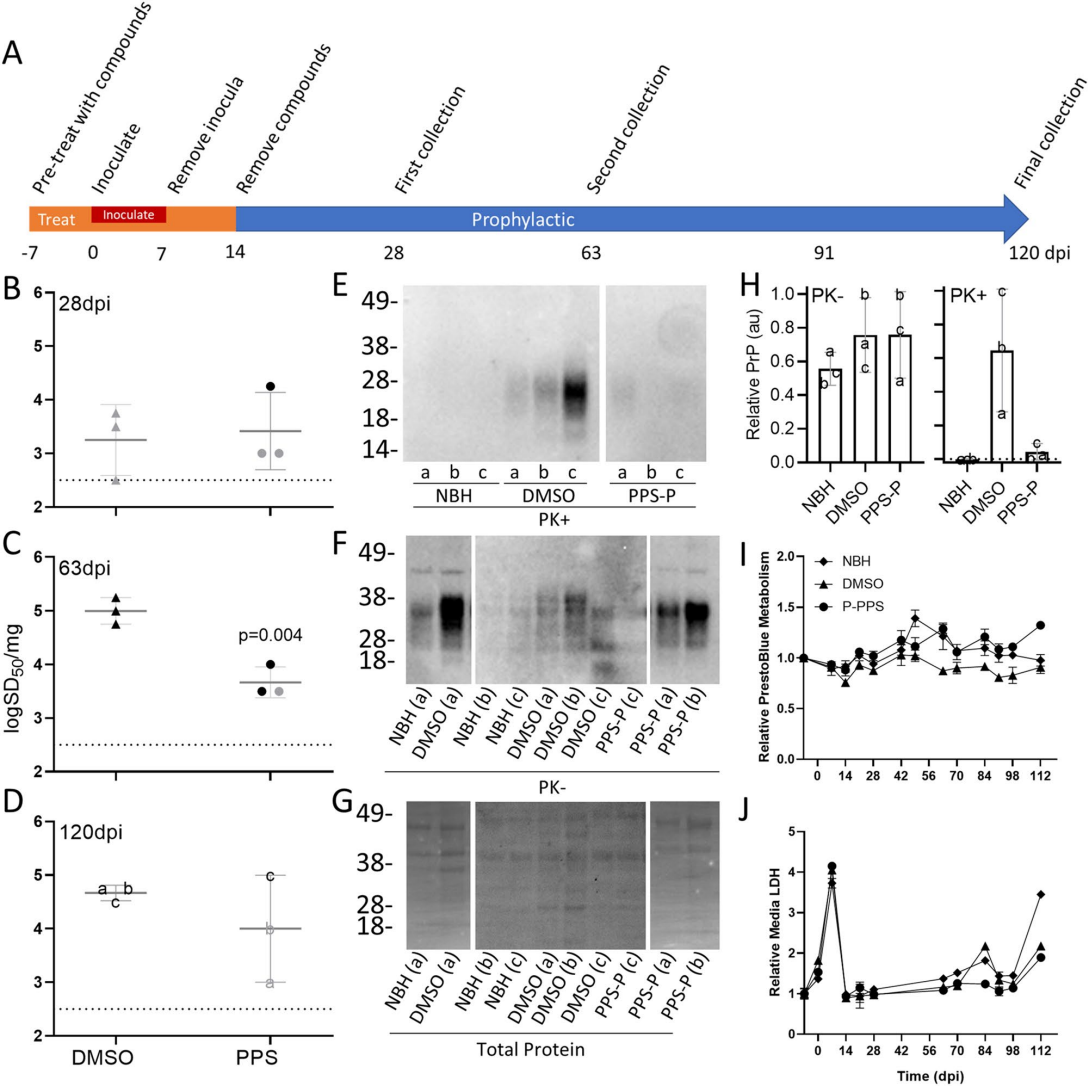
Prophylactic treatment reduces prion seeding activity. **(A)** A schematic representation of infection and treatment timeline. COs were prophylactically exposed to PPS (PPS-P) or DMSO (vehicle) from 7 days before (− 7) to 14 days after the day of inoculation (including during the inoculation). The COs were infected through the addition of the inoculum into the media followed by 7 days of incubation. **(B–D)** Log SD_50_s per mg tissue of RT-QuIC seeding activity from COs treated prophylactically with PPS or exposed to DMSO and collected at 28 **(B)**, 63 **(C)**, or 120 dpi **(D)**. Each data point represents a single organoid with mean + /− SD from three COs represented. The dashed line indicates the lower detection limit of the assay. Data points colored gray indicate an over-estimation of log SD_50_ values as these samples did not have a tested dilution where all replicate wells had positive reactions. Western blots showing protease-resistant PrP (PK + ; **E**), total PrP (PK−; **F**), and total protein stain for the PK− blot **(G)** for 120 dpi organoids. Letters a, b, and c denote individual organoids and correspond with those shown in **(D–H)**. Samples were run over several blots and spliced for simplicity. Uncropped blots can be found in Supplementary Fig. S4. To adjust for the use of multiple blots samples were quantified **(H)**. Total PrP, relative to the total protein, was normalized to the maximum value (**H**; PK−). Proteinase K digested samples were quantified by subtracting the baseline of the average NBH values and then normalized to the maximum value (**H**; PK +). The dashed line denotes “0”. Error bars represent standard deviation. **(I)** PrestoBlue analyses were performed to monitor the cellular metabolism and viability of the COs over time. **(J)** In parallel, cultures were monitored for cellular breakdown in COs by measuring LDH levels in the media. The same three organoids were monitored throughout the study. Each value is relative to day − 7 (before treatment or infection). Error bars represent standard deviation from triplicate reading of three pooled organoids. DMSO and PPS labels refer to sCJD infected organoids. NBH was similarly treated with DMSO.

Our initial goal was to assess whether COs could be used as a model analogous to prophylactic drug treatments for prion disease. PPS treatment was not toxic to the COs (Supplementary Fig. S1A) and did not induce major changes in the expression (Supplementary Fig. S1B and C) or localization of PrP^C^ (Supplementary Fig. S1D), as has been previously reported in cell culture^37,44^ and animal models^45^. Therefore, the COs were incubated in 3 µg/ml of PPS in media or the DMSO vehicle for 21 days (day -7 to day 14 Fig. 1A). Following the initial 7 days of treatments, the COs were inoculated with 0.1% sCJD MV2 brain homogenate or normal brain homogenate (NBH) in media containing the treatment compound or DMSO for 1 week (day 0 to day 7). The COs were then transferred to new vessels in fresh media containing PPS or DMSO. After the final 7-day incubation the treatments were removed through a complete media change and change of vessel.

Infection of the COs was monitored through prion seeding activity. Samples were collected at 28, 63, and 120 dpi and analyzed by RT-QuIC for seeding dose 50 s (SD_50_s), analogous to lethal dose 50 (LD_50_) in animal bioassays^46^, as an estimate of the concentration of seeding activity units giving positive reactions in 50% of replicate reactions (Fig. 1B–D; see “Methods”). At 28 dpi, 2 weeks after the treatments had been removed from the media, logSD_50_s per mg of tissue were measured from CO homogenates. At this timepoint the DMSO and PPS treated COs had similar SD_50_s (Fig. 1B). The NBH treated organoids did not show any seeding activity so logSD_50_s could not be calculated. The seeding activity in the sCJD infected organoids exposed to DMSO increased by nearly 100-fold by 63 dpi (Fig. 1C) which persisted through 120 dpi (Fig. 1D), confirming de novo production of seeding activity and bona fide infection. In contrast, the PPS treated COs had tenfold less seeding activity than the DMSO group at 63 dpi (49 days after the removal of PPS) (Fig. 1C). While no longer statistically significant, the PPS treated COs continued to show a trend of weaker seeding activity compared to the DMSO group at 120 dpi (106 days after the removal of PPS) (Fig. 1D).

After the final collection at 120 dpi, COs were probed by western blot for protease-resistant PrP, a hallmark of prion disease, and for histological and immunohistochemical signs of disease. The sCJD infected COs exposed to the DMSO vehicle showed protease-resistant PrP in all three COs analyzed (Fig. 1E,H, DMSO), albeit differing from the monoglycosylation-dominant banding pattern typical of sCJD towards di-glycosylated-dominant as previously reported^27^. The levels of total PrP relative to the total protein varied somewhat between organoids with the CJD inoculated organoids showing slightly higher levels, likely due to PrP^Sc^ accumulation (Fig. 1F–H). Immunohistochemical staining for total PrP revealed diffusely spread staining around the periphery in all CO groups (Supplementary Fig. S2). However, internal areas of coarse, punctate, granular PrP staining were displayed in all three CO’s inoculated with CJD and exposed to a DMSO treatment (Supplementary Fig. S2 inset, magenta arrowheads) similar to the sCJD-like staining pattern previously reported^27^.

In contrast, the PPS treated COs showed little protease-resistant PrP and only in two of the three COs tested (Fig. 1E,H, PPS-P). The sCJD-like PrP staining pattern observed by immunohistochemical staining in the COs exposed to DMSO was not observed in any of the PPS treated or NBH inoculated COs (Supplementary Fig. S2). Hematoxylin and eosin (H&E) staining for vacuolation, however, was variable and indistinguishable between control and treatment groups (Supplementary Fig. S3) as was reported previously^27^.

To ensure the changes in seeding activity were a result of the treatment, cultures were monitored for metabolic changes, through a resazurin-based PrestoBlue assay (Fig. 1I), and necrosis, as measured through lactate dehydrogenate (LDH) levels in the media (Fig. 1J). Consistent with previous reports^27^, the metabolism and LDH levels in each group remained steady for the duration of the study until the end stages where age related cell death was observed and the experiment study was concluded (Fig. 1I,J). A spike in LDH readings was observed during the inoculation (Fig. 1J), likely contributed by the addition of the brain homogenate as it was observed in both the sCJD and normal brain homogenate (NBH) inoculated organoids; however, organoid metabolism was unaffected (Fig. 1I).

### Therapeutic treatment of COs with PPS

**Figure 2:**
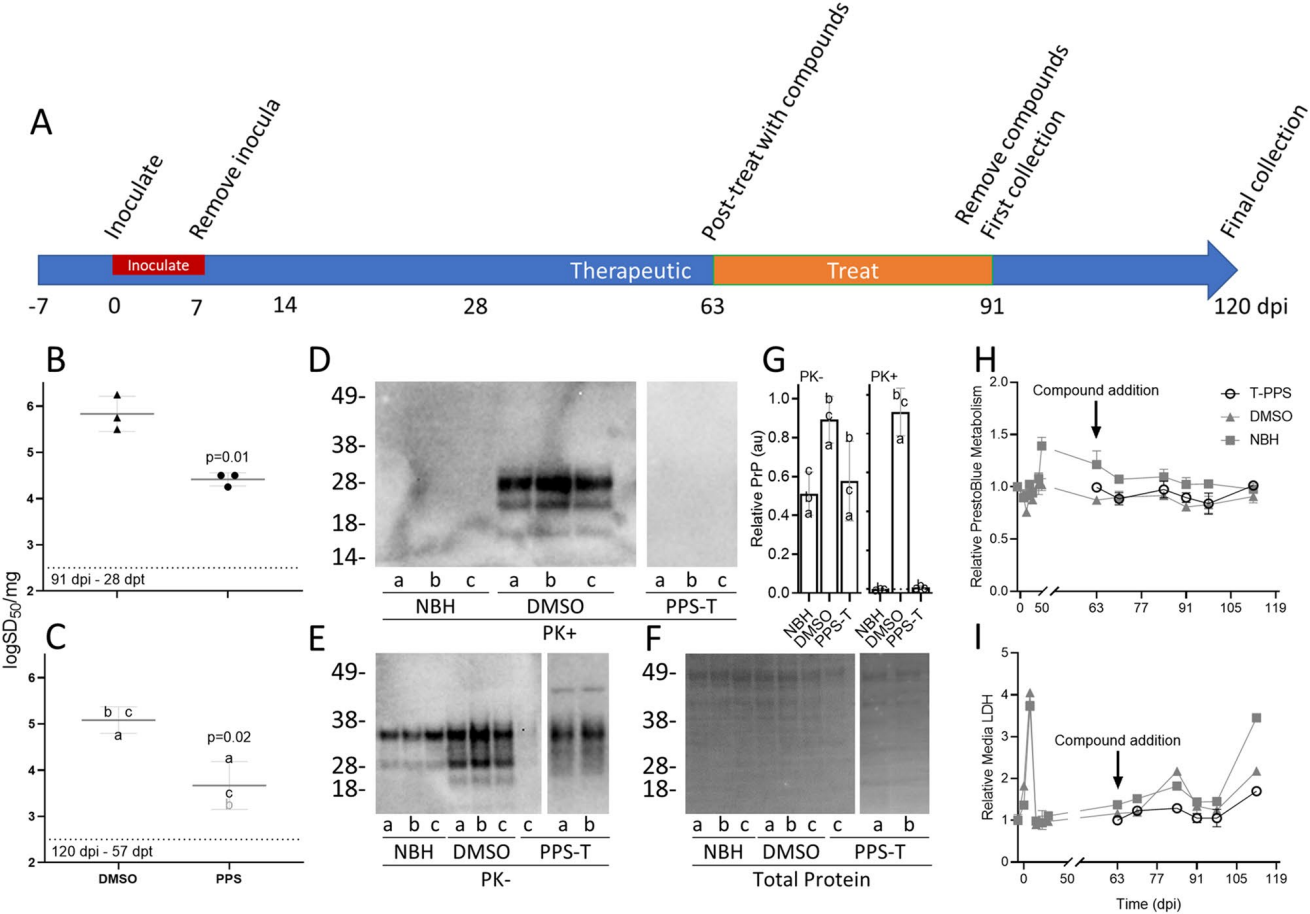
Therapeutic treatment reduces prion seeding activity. **(A)** A schematic representation of infection and treatment timeline. COs were inoculated with sCJD brain homogenate or NBH and therapeutically exposed to DMSO (vehicle) or PPS (PPS-T) from 63 to 91 DPI. **(B,C)** Log SD_50_s per mg tissue of RT-QuIC seeding activity from COs treated therapeutically with DMSO or PPS and collected at 91 **(B)**, or 120 dpi **(C)**. Each data point represents a single organoid with mean + /− SD from three COs represented. The dashed line indicates the lower detection limit of the assay. Data points colored gray indicate an over-estimation of log SD_50_ values as these samples did not have a tested dilution where all replicate wells had positive reactions. Western blots showing protease-resistant PrP (PK + ; **D**), total PrP (PK−; **E**), and total protein stain for the PK− blot **(F)** for 120 dpi organoids. Letters a, b, and c denote individual organoids. Samples were run over several blots and spliced for simplicity. Uncropped blots can be found in Supplementary Fig. S4. To adjust for the use of multiple blots samples were quantified **(G)**. Total PrP, relative to the total protein, was normalized to the maximum value (**G**; PK−). Proteinase K digested samples were quantified by subtracting the baseline of the average NBH values and then normalized to the maximum value (G; PK +). The dashed line denotes “0”. Error bars represent standard deviation. **(E)** PrestoBlue analyses were performed to monitor the cellular metabolism and viability of the COs over time. **(F)** In parallel, cultures were monitored for cellular breakdown in COs by measuring LDH levels in the media. The same three organoids were monitored throughout the study starting just prior to the addition of PPS. The control values (gray) are the same traces from Fig. 1. Error bars represent standard deviation from triplicate reading of three pooled organoids. DMSO and PPS represent treatments on sCJD infected organoids. NBH was similarly treated with DMSO.

Our second goal was to test whether COs could be used as a model of therapeutic drug treatments in patients already displaying signs of disease. COs were inoculated alongside the control sCJD and NBH organoids from the pretreatment group (Fig. 2A) and allowed to progress for 63 days. After 63 dpi de novo propagation of PrP^Sc^ had resulted in the accumulation of ~ 5 logs of seeding activity (Fig. 1C, DMSO). The sCJD infected COs were then cultured in the presence of DMSO or PPS for 28 days (63-91dpi) prior to collection (91 dpi [28 dpt], Fig. 2A) and assessed by RT-QuIC for seeding activity. COs exposed to the DMSO vehicle showed nearly a tenfold increase in seeding activity over this time while PPS treated COs showed nearly a tenfold decrease in seeding activity (Fig. 2B). The remaining COs were allowed a recovery period of another 29 days without DMSO or PPS before the final collection to assess whether this treatment would “cure” the infection or if continued treatment might be necessary. The sCJD infected organoids (120 dpi [57 dpt]) exposed to DMSO maintained a consistent level of seeding activity over this time (Fig. 2C). COs that had been treated with PPS continued to show a significant reduction compared to DMSO group (Fig. 2C). Additionally, the 120 dpi PPS treated COs did not show any protease-resistant PrP (Fig. 2D,G, PPS-T) or sCJD-like immunohistochemical PrP staining (Supplementary Fig. S2, PPS-T) like was observed in the 120 dpi COs exposed to the DMSO vehicle. Total PrP levels relative to total protein were variable between organoids (Fig. 2E–G), however, the DMSO treated organoids inoculated with CJD again showed higher total PrP levels than the other groups, likely due to PrP^Sc^ accumulation (Fig. 2E–G). No changes in metabolism (Fig. 2H, open symbols) or LDH (Fig. 2I, open symbols) were observed across the duration of the study, again suggesting that the changes in seeding activity were a result of the treatment.

## Discussion

Herein we have assessed the practicality of a human CO model of sCJD for evaluating the effectiveness of putative drugs in inhibiting prion accumulation. The human COs demonstrated utility in two different paradigms mimicking a prophylactic or therapeutic treatment. These two paradigms are designed to address treatments for genetic carriers or accidental exposures^9–12^, or a symptomatic patient, such as with sCJD, respectively. While a drug candidate would ideally be effective in both scenarios, some drug candidates may only show efficacy with pretreatment but may be unable to sufficiently slow or eliminate the disease once it has progressed and tissue damage has occurred. On the other hand, some candidates may be effective at acutely knocking down prion deposition and slowing disease progression but may be unsuitable for long term treatments as a prophylactic in an otherwise healthy patient.

In the prophylactic model we found that PPS was able to demonstrate a slowing in the accumulation of prion seeding activity and deposition of protease resistant and aggregated PrP (Fig. 1 and Supplementary Fig. S2). In the therapeutic model we show that PPS was able to reduce the level of prion seeding activity and prevent or reduce the deposition of protease resistant and aggreagated PrP (Fig. 2 and Supplementary Fig. S2). In this initial study we chose a treatment window rather than a sustained treatment in order to assess whether we could determine if infection was cured or would rebound after removal of the treatment. Since we never observed a complete loss of prion seeding activity in either treatment group during the experimental window it is not surprising that we did observe a rebound under these conditions. A longer, sustained treatment, as would be performed in a clinical setting, would likely have a more robust effect and potentially cure the COs. Additionally, an apparent reduction in seeding activity, albeit not statistically significant, was observed in the DMSO groups at their final time points compared with their previous time points. This is likely due, in part, to a cumulative effect of the extended DMSO treatments. DMSO has been shown to interfere with PrP^Sc^ formation^47–49^ and prolonged exposure to DMSO might result in a decreased seeding capacity. However, this effect would be equivalent between the DMSO and treatment groups and therefore, while important to monitor, it would not diminish the significance of any treatment effects.

Whilst not high-throughput as compared with secondary cell line screening, a number of different test paradigms (e.g. different doses, timeframes, prophylactic or therapeutic regimens) can be done prior to or instead of in vivo assays. This supports the use of COs as a model for assessing neurotoxicity and efficacy of drug candidates and offers an ethical reduction in animal usage. Although this initial study utilized only a small number of organoids per collection, it is simple to scale up and include increased numbers of COs per condition, multiple doses and/or timeframes, and other parameters with minimal extra resources. Indeed, higher numbers of organoids would be required to fully evaluate the efficacy of a potential drug candidate.

As a model for screening therapeutic candidates for prion disease, COs are not without their limitations. We acknowledge the lack of vascularization may limit penetration of the drug candidates being tested, which may mask the effect of some treatments. Additionally, COs generated here do not contain non-neuronally derived cells such as microglia or epithelial cells, and, despite displaying a high degree structural organization, are not organized into specific brain regions. The lack of organized brain regions is likely the reason that the cytopathic signs typically associated with prion diseases, such as astrogliosis and spongiosis, tend to be indistinguishable from control COs since lesion profiles are associated with specific brain structures. Often diffuse PrP staining can be observed in the COs which does not appear to be disease associated but can make it difficult to identify disease associated PrP deposits. However, coupling this model with highly sensitive RT-QuIC assays for prion seeding activity^46,50^ has allowed us to bypass many of the above-mentioned weaknesses by providing a means for robust, high-throughput, quantitative detection of disease associated, seeding competent PrP early in infection. This allows us to monitor the efficacy of a drug treatment over time long before PrP deposition or cytopathic changes would be observed.

While decades of work have yet to identify an effective treatment for prion diseases^51^, promising approaches continue to be developed. For example, antisense oligonucleotide treatments^52^ have recently been shown to be effective in prolonging survival time in mouse models, and novel compounds^53,54^ have shown promise in in vitro and in silico models. Currently, the CO model is not a replacement for animal models, however, COs could be used to substitute certain studies in animals and will complement the existing model repertoire, providing a strong indicator of treatment efficacy in a human system. The availability of a human CO model promises to be a much-needed next step towards advancing these strategies towards screening and testing potential therapeutics for human prion disease.

## Methods

### Human cerebral organoid generation and culture conditions

KYOU-DXR0109B (ACS-1023; ATCC) hu-iPSCs^27^ were used to generate the COs. COs were generated and maintained as described in^55^ with modifications described in^27^. COs were maintained in cerebral organoid media (1 × glutamax, 1 × penicillin–streptomycin solution, 0.5 × non-essential amino acids, 0.5% [v/v] N2, 1 µl/4 ml insulin, and 1 µl/286 ml 2-Merceptoethanol in 1:1 Neurobasal:DME-F12 medium) with 1% (v/v) B12).

### Prion infections and treatment of human cerebral organoids

COs were cultured for ~ 5 months before infecting as per our previously described protocol^27^. To match previous reports, the COs were heterozygous at codon 129 (129MV) and the inocula used was from a 129MV donor. The PrP subtype 2 was used based on our previous work that showed a faster progression with an increased level of PrP^Sc^ accumulation in the MV2 subtype^27^. For prophylactic testing COs were exposed to sodium pentosan polysulfate (PPS; Sigma)^37^ or a dimethyl sulfoxide (DMSO) vehicle for the seven days prior to infection, the 7 days during infection, and for one week after removal of the inocula (Fig. 1A). The inocula was diluted 1:1 after 24 h and again after 4 days to prevent excess toxicity of the homogenate. The logSD_50_s per mg of brain for the inocula used were 7.20 for the DMSO group and 7.45 for the PPS group. To assess the therapeutic effect of PPS, COs were taken from the DMSO prophylactic group at 61 dpi and exposed to PPS or a DMSO vehicle for one month. (Fig. 2A). While DMSO is not required to solubilize PPS, many drug candidates do require it. Additionally, DMSO has been shown to inhibit PrP^Sc^ formation and delay disease in vivo^47–49^. Therefore, we tested PPS in the background of 0.2% DMSO to ensure that a treatment effect could be observed even in the presence of DMSO. NBH controls received the same DMSO treatment. Brain tissue used in this study was obtained on autopsy and approved by the ethics committee at Istituto Superiore di Sanità (Italy), which is recognized by the Office for Human Research Protections of the U.S. Department of Health and Human Services. Informed consent for participation in research was obtained from human subjects or legal representatives in accordance with the Declaration of Helsinki and the Additional Protocol to the Convention on Human Rights and Biomedicine, concerning Biomedical Research. All patient data and samples were coded and handled according to NIH guidelines to protect patient identities in accordance with the National Institutes of Health Office of Human Subjects Research Protections.

### Prestoblue analysis

PrestoBlue (Thermofisher) metabolism was measured as per the manufacturer’s instructions and as described in^27^ on the same 3 pooled organoids from each group for the duration of the study. Prestoblue fluorescence was measured at 560 nm excitation and 590 nm emission in a ClarioStar plate reader (BMG).

### Lactate dehydrogenase (LDH) analysis

Extracellular LDH was measured using cytotoxicity detection kit plus [LDH] (Roche) as per manufacturer’s instructions and described in^27^. Results shown are obtained from the same 3 organoids as used for Prestoblue analysis. The assay was performed in a ClarioSTAR plate reader (BMG) measuring absorbance at 492 nm. For the assessment of toxicity by LDH analysis, following a 4-day treatment 3 COs were measured independently per group for media LDH and total (lysed) LDH as per manufacturer’s instructions. Percent cytotoxicity is expressed as media LDH/total LDH times 100.

### RT-QuIC

Real-time quaking-induced conversion (RT-QuIC) assays were performed as previously reported^27,56^. COs were homogenized to 10% (w/v) in phosphate buffered saline (PBS) and then serially diluted by 10 folds in 0.1% SDS/PBS/N2. Each well was loaded with 49 μL reaction mix (10 mM phosphate buffer [pH 7.4], 300 mM NaCl, 0.1 mg/mL hamster recombinant PrP 90–231, 10 μM thioflavin T, and 1 mM ethylenediaminetetraacetic acid tetrasodium salt) and seeded with 1 μL of CO dilution in 0.1% sodium dodecyl sulfate (SDS)/PBS/N2 for a final SDS concentration of 0.002% in 384-well plate (Nunc). Each sample was tested in quadruplicate. Reaction wells were considered positive when they exceeded a threshold of 10% of the maximum average value from quadruplicate reactions on each plate within the 90-h time cutoff. LogSD_50_s were calculated using Spearman-Kärber analyses^57^ to provide estimates of the concentrations of seeding activity units giving positive reactions in 50% of replicate reactions, i.e., the 50% “seeding doses” or SD_50_’s as previously described^27,46^. Three organoids were tested per condition. Data was plotted and analyzed using GraphPad (Prism). Statistical calculations of p-values were performed using an unpaired t-test with Welch’s correction.

### Proteinase-K digests and western blotting

COs were homogenized using a motorized pestle to 10% (w/v) in PBS. Homogenates were incubated for 1 h at 37 °C under 400 rpm shaking with 5 μg/ml Proteinase K and 1% Sarkosyl. The reactions were terminated by addition of 1 μM Pefabloc for 5 min at 4 °C. Samples were then mixed with 4X Bolt LDS sample buffer (Invitrogen) containing 8% β-mercaptoethanol and boiled for 10 min. Five μL equivalents of undigested or 20 μL equivalents of Proteinase K digested samples were run on Bolt 4–12% Bis–Tris gels (Invitrogen) and transferred to PVDF membranes using the iBlot 2 transfer system (Invitrogen). PrP was detected using the 3F4 antibody (Millipore) at a 1:10,000 dilution and visualized using ECL Select (Amersham) on the iBright imaging system (Invitrogen). Total protein was visualized by Coomassie blue staining. Quantitation was performed using ImageJ (FIJI) software (NIH).

### Histochemistry and immunohistochemistry

For consistency we maintained the same histology procedures, antigen retrieval, hematoxylin–eosin (H&E) and protein staining as described previously for the organoid cultures^27,58^. Antibody staining used monoclonal antibody 6H4 (Prionics) at a 1:6000 dilution for PrP. All histopathology slides were analyzed by observers blinded to the inoculation groups using Aperio Imagescope software.

### Immunofluorescence

Cerebral organoids treated with PPS or exposed to the DMSO vehicle were fixed for 24 h in 10% neutral buffered formalin, washed three times in 1× PBS for 15 min and equilibrated overnight in PBS with 30% sucrose for cryoprotection. Whole organoids were then embedded in OCT, frozen to -20℃ and sectioned at 10 µm on a Leica 3050s cryostat (Leica). Sections were blocked with 5% normal donkey serum, 0.1% Triton-X-100 and 0.3 M Glycine in PBS for 30 min at RT followed by overnight incubation with SAF32 primary antibody (1:250, Cayman Chemicals) at 4 ℃. The SAF32 epitope resides in the amino-terminal octareapeat region of PrP (amino acids ~ 51–91). PPS has been identified to bind to this same region^37,59,60^ resulting in decreased antibody binding in the PPS treated samples and an apparent lower PrP signal. The primary antibody was visualized by donkey anti-mouse AF647 (1:1000, ThermoFischer) secondary antibody incubated for 1 h at RT. At the same time, 488 Phalloidin (1:40, ThermoFischer) was applied to visualize cellular actin. Hoechst 33342 (1:2000 of a 20 mg/mL solution, Tocris) was applied to visualize cell nuclei. Slides were cover slipped with Prolong Gold (Molecular Probes) and images taken using a Zeiss 710 LSM (Carl Zeiss) with a Plan Apochromat 63x/1.4 numerical aperture oil-immersion objective. Image deconvolution was performed using Huygens Essential v.19.04 (SVI) and figures built using Imaris v.8.4.1 (Bitplane) and Adobe Photoshop CC2019 (Adobe).

## Supplementary Information

Supplementary Figures.

## Data Availability

The datasets generated and/or analyzed during the current study for the purpose of this article are available from the corresponding author on reasonable request.
