# Supplementary material for: Human cerebral organoids as a therapeutic drug screening model for Creutzfeldt–Jakob disease
Source: Sci Rep. 2021 Mar 9;11:5165. doi: 10.1038/s41598-021-84689-6 (PMC7943797; doi:10.1038/s41598-021-84689-6)
Supplement: Supplementary file 1 — Supplementary Figures. [file 41598_2021_84689_MOESM1_ESM.docx]

Supplementary information for:

Human cerebral organoids as a therapeutic drug screening model for Creutzfeldt-Jakob Disease

Bradley R. Groveman^1^†, Natalia C. Ferreira^1^†, Simote T. Foliaki^1^, Ryan Walters^1^, Clayton W. Winkler^1^, Brent Race^1^, Andrew G. Hughson^1^, Gianluigi Zanusso^2^, and Cathryn L. Haigh^1^*

^1^Laboratory of Persistent Viral Diseases, National Institute of Allergy and Infectious Diseases, Division of Intramural Research, Rocky Mountain Laboratories, National Institutes of Health, Hamilton, MT 59840, USA.

^2^Department of Neurosciences, Biomedicine and Movement Sciences, University of Verona, 37134 Verona, Italy

†Equal contribution

*Correspondence: cathryn.haigh@nih.gov


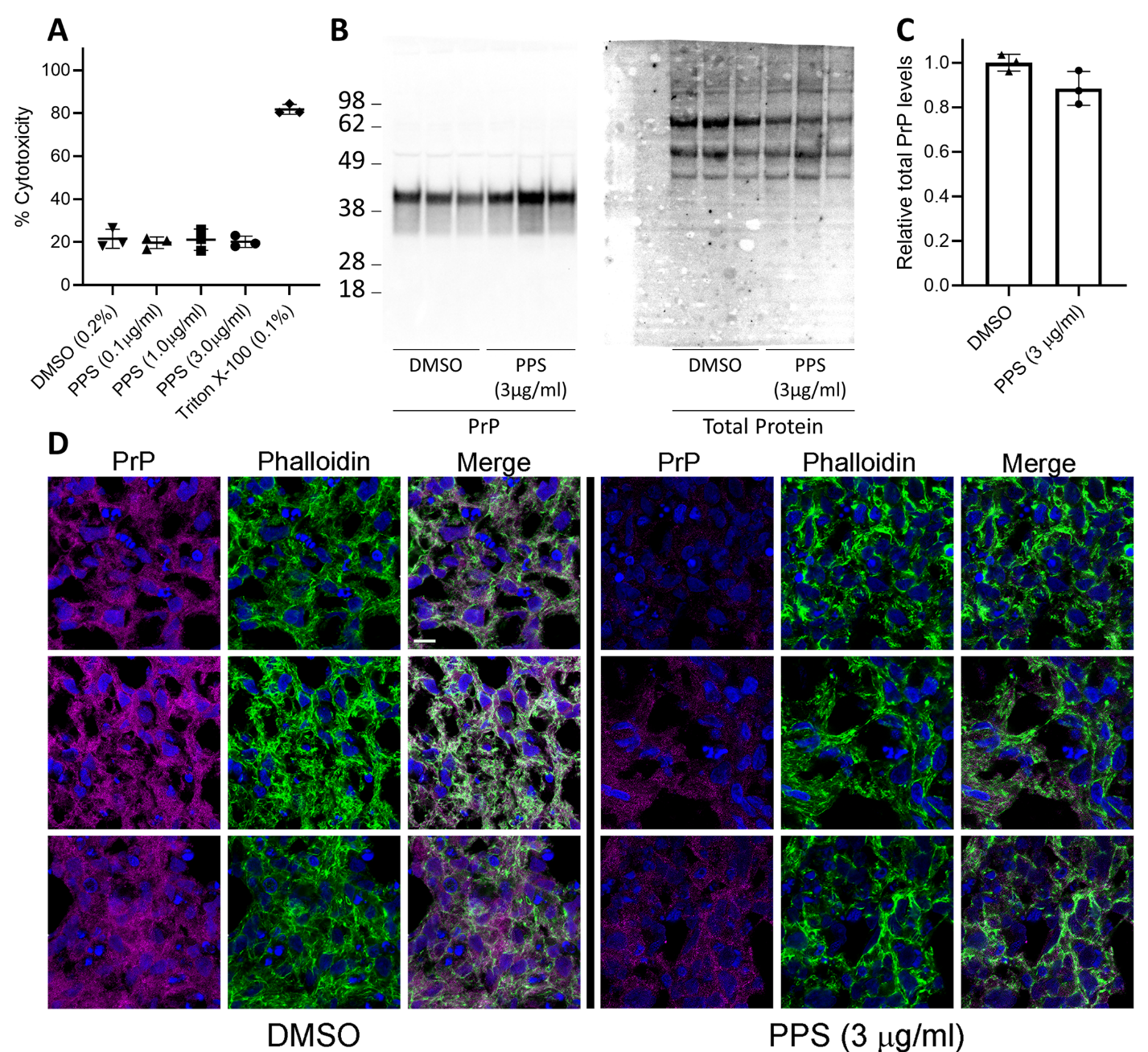


**Supplementary Figure S1: Characterization of PPS treatment.** A) Cytotoxicity (media LDH as a percent of total LDH), was assessed following a four-day treatment with 0.1 µg/mL, 1.0 µg/mL, and 3.0 µg/mL PPS (N=3). Mean and standard deviation is displayed. B-C) Cells were assessed for PrP expression by western blot following a four-day treatment with PPS (3 µg/mL). B) Organoid lysates were blotted for total PrP using the 3F4 antibody (PrP) and normalized to total protein (Total Protein). C) PrP expression was quantified from the western blot and calculated relative to total protein. Values are displayed relative to DMSO (N=3). Each data point represents an individual organoid. D) PrP localization in the cells of the COs following a four-day treatment with PPS. Cells were stained for PrP (magenta; SAF32 antibody), Phalloidin (green) and DAPI (blue). Colocalization is shown in white (Merge). PrP levels artificially appear lower in the PPS treated samples due to PPS interference with the SAF32 epitope (see methods). Scale bars indicate 10 µm.


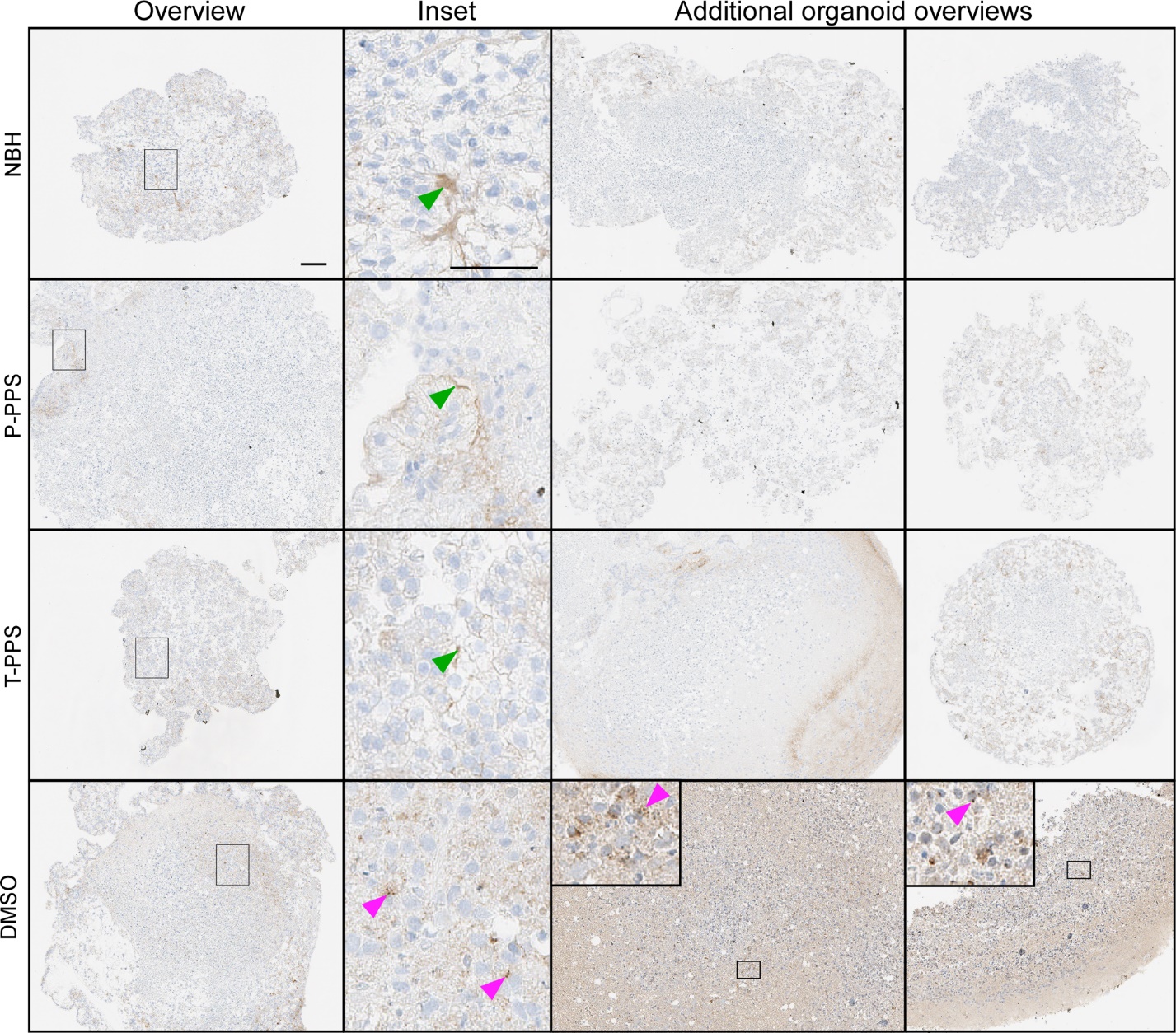


**Supplementary Figure S2: PrP deposition in 120dpi COs.** Organoid overviews and high magnification insets display diffusely spread PrP staining in nearly all organoids (green arrows). Only the DMSO treated CJD organoids display some areas of coarse, punctate, granular PrP staining (magenta arrows). P-PPS indicates prophylactic treatment while T-PPS indicates therapeutic treatment. N=3 for each group. Due to technical issues the second and third DMSO treated organoids were processed separately and have a higher level of background stain from the secondary antibody. Scale bars represent 100µm in the overview panels and 50µm for all of the higher magnification insets.


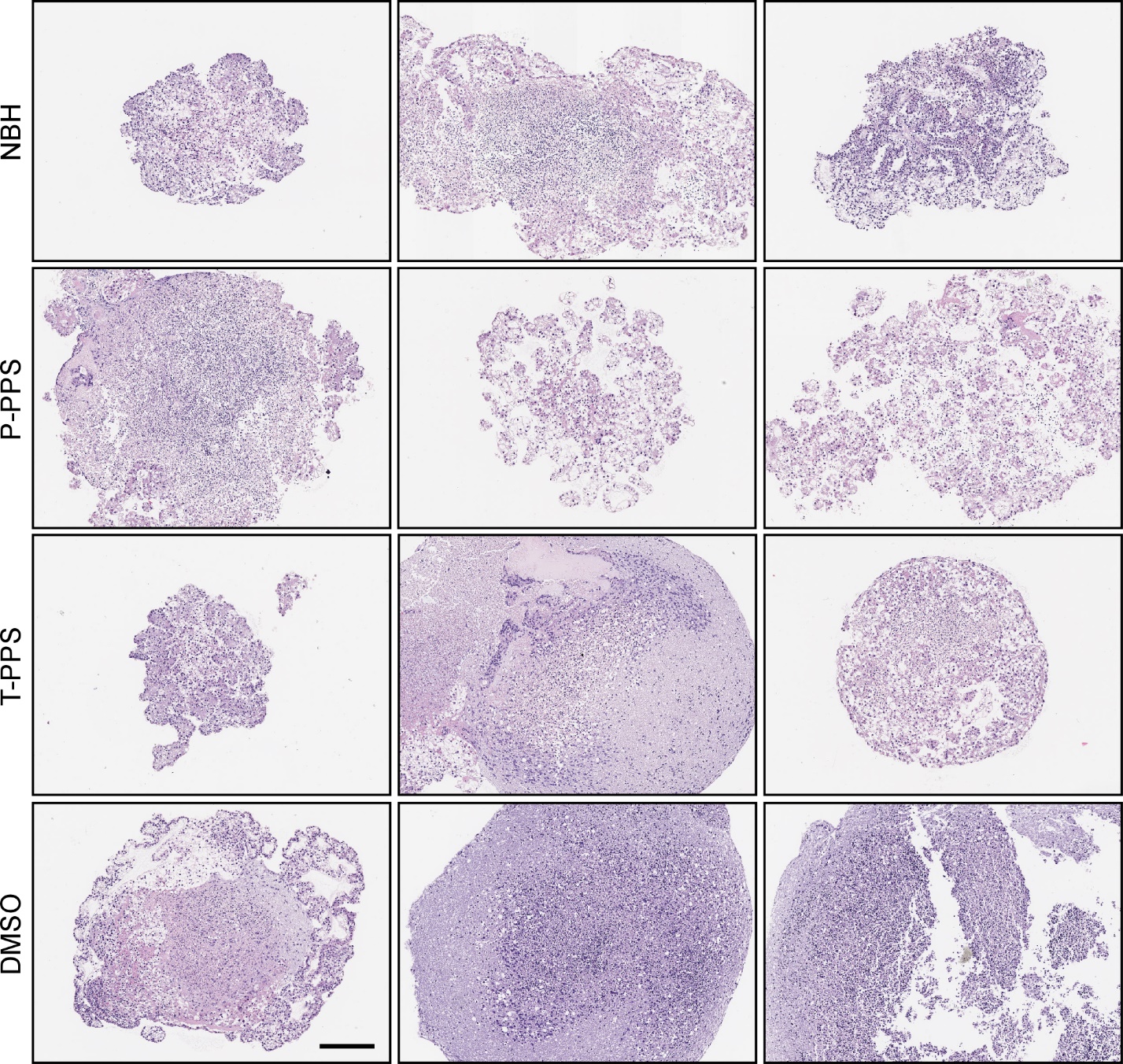


**Supplementary Figure S3: H&E staining in all 120dpi COs.** No disease-specific spongiosis is observed in any of the organoids. P-PPS indicates prophylactic treatment while T-PPS indicates therapeutic treatment. N=3 for each group. Due to technical issues the second and third DMSO treated organoids were processed separately. Scale bar represents 250µm.


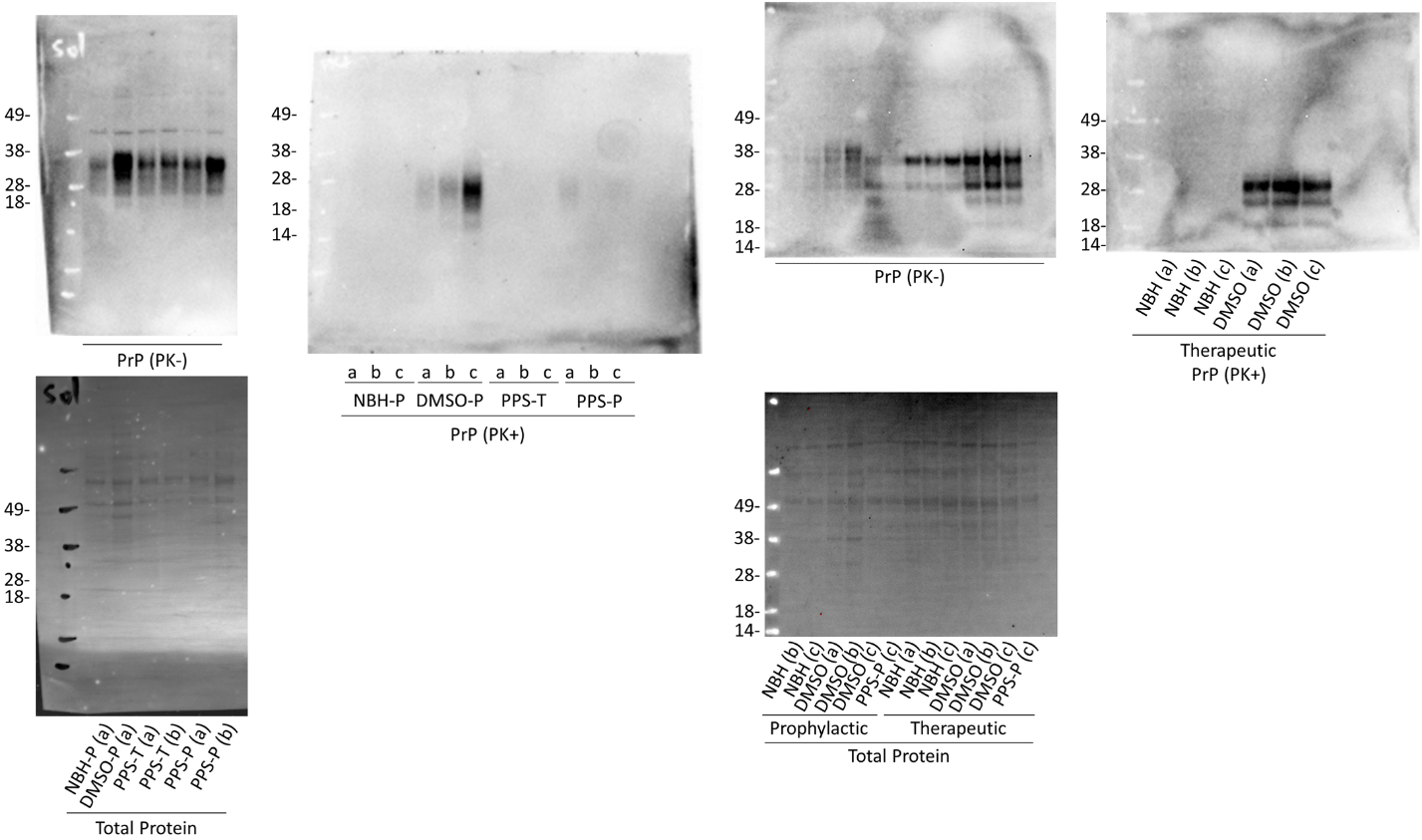


**Supplementary Figure S4: Uncropped western blot images from Figures 1 and 2.** Membranes were probed for PrP with the 3F4 antibody (PrP) or stained for total protein with (PK+) or without (PK-) Proteinase K digestion. Prophylactic (-P) samples were shown in Fig. 1. Therapeutic (-T) samples were shown in Fig. 2.
